# Biological and Analytical Perspectives on D-Amino Acids in Cancer Diagnosis and Therapy

**DOI:** 10.3390/ph18050705

**Published:** 2025-05-09

**Authors:** Alina Uifălean, Maria Iacobescu, Liana Claudia Salanță, Simona Codruța Hegheş, Radu-Cristian Moldovan, Cristina-Adela Iuga

**Affiliations:** 1 Department of Pharmaceutical Analysis, Faculty of Pharmacy, “Iuliu Hațieganu” University of Medicine and Pharmacy, Louis Pasteur Street 6, 400349 Cluj-Napoca, Romania; alina.uifalean@umfcluj.ro (A.U.); cmaier@umfcluj.ro (S.C.H.); cristina.iuga@medfuture.ro (C.-A.I.); 2 Department of Personalized Medicine and Rare Diseases, MEDFUTURE—Institute for Biomedical Research, “Iuliu Hațieganu” University of Medicine and Pharmacy, Louis Pasteur Street 6, 400349 Cluj-Napoca, Romania; maria.iacobescu@medfuture.ro; 3 Department of Food Science, Faculty of Food Science and Technology, University of Agricultural Sciences and Veterinary Medicine, Calea Mănăștur Street 3-5, 400372 Cluj-Napoca, Romania; liana.salanta@usamvcluj.ro

**Keywords:** D-amino acids, chirality, enantiomer separation, cancer, biomarker, early diagnosis

## Abstract

For a long time, D-amino acids remained unexplored in mammalian physiology. The technological advances in enantioseparation over the past 50 years have revealed that D-amino acids not only exist in human tissues and fluids but also play important roles in neurotransmission, immune regulation, and cellular proliferation. The present review provides a comprehensive assessment of the role of D-amino acids in cancer, including their endogenous and exogenous production pathways, along with the analytical methodologies used for detection and quantification, from liquid chromatography to biosensors. These methods have underlined how altered levels of D-amino acids can be helpful in early detection, progression, or response to treatment in several malignancies, including gastric, hepatic, colorectal, or breast cancer. The present review also explores how manipulation of D-amino acids can regulate cell proliferation, their mechanisms in cancer regulation, including the modulation of *N*-methyl-D-aspartate (NMDA) receptors and the production of hydrogen sulphide (H_2_S), and the role of specific D-amino acids in cancer onset, immune defence, and protection against chemotherapy-induced toxicity. Finally, several underexplored research directions are outlined, such as potential correlations with gut microbiota composition, the impact of processed food consumption, and the integration of multiomics strategies.

## 1. Introduction

Amino acids are fundamental molecules for all living organisms. They act as building blocks for proteins, necessary for nearly all biological functions, from protein synthesis to numerous metabolic processes such as nitrogen balance, energy production, nucleotide biosynthesis, and cell signalling. Moreover, they are involved in enzyme and hormone synthesis, influencing immune function and neurotransmission [1].

Apart from glycine, the α-carbon of the 19 proteinogenic amino acids is linked to four different substituents. This carbon represents a stereogenic or a chiral centre, allowing amino acids to exist as L- or D-enantiomers, which are mirror images (‘right-handed’ and ‘left-handed’), with different spatial arrangements of their substituents [2]. These relatively minor changes in the stereochemistry of amino acids are very important, as they can trigger physicochemical and functional changes in the proteins in which these amino acids are assembled [3].

For a long time, in eukaryotic cells, only L-amino acids were thought to participate in protein synthesis. Therefore, D-amino acids were considered irrelevant or accidental byproducts of metabolism. The presence of D-amino acids in cells was first documented in 1939 by Kögl et al., when D-Glu was extracted from tumour proteins [4]. Nowadays, it is known that not only free D-amino acids are present in various eukaryotic organisms, but also several D-amino acid-containing peptides [5,6].

Developments in the selectivity and sensitivity of analytical techniques have permitted the identification of D-amino acids in various biological samples, including the brain, kidney, liver, blood, urine, saliva, and cerebrospinal fluid of rodents and humans [1]. D-Ser, D-Asp, and D-Ala are the most abundant D-amino acids in mammals, with the highest concentrations found in the hippocampus, pituitary gland, and cerebrum [7]. These D-amino acids were also the most studied ones, mainly due to their role as agonists for *N*-methyl-D-aspartate (NMDA) receptors, implicated in synaptic plasticity, learning, and memory [1,8]. Predominantly D-Ser was explored as a potential biomarker and/or therapeutic agent for neurological and psychiatric conditions such as amyotrophic lateral sclerosis, schizophrenia, and Alzheimer’s disease [9]. Moreover, when associated with D-Pro, D-Ser represented a reliable marker of early cognitive decline, severe depressive disorders, and post-traumatic stress disorders [10]. In addition to this, D-amino acids were also investigated in other physiological processes, including cell signalling, antimicrobial activity, and glucose homeostasis [1,11,12].

The physiological ratio of L- and D-amino acids can be altered by various biological factors such as mutations or deficiencies in chiral-selective enzymes, genetic mutations, or gut dysbiosis [13,14], as well as chemical factors like free radical accumulation, exposure to toxins, and pollutants [15]. These alterations result in racemization and enantioselective accumulation in human tissues.

Also, these factors are recognized as pathogenic triggers in various disorders, such as Alzheimer’s disease, schizophrenia, chronic kidney disease, metabolic conditions, and cancers [6,12,16,17,18,19]. In addition, modulation of D-amino acids or their associated pathways could represent potential therapeutic options for novel, targeted therapies [10,18]. As an example, a recent LC-MS analysis of urine samples from colorectal cancer (CRC) patients and healthy volunteers showed that D-amino acids, specifically D-Ala, D-Gln, D-Ser, and D-alpha-aminobutyric acid (D-AABA), can represent potential biomarkers for CRC, being able to distinguish CRC patients from healthy controls with a 95.2–96.7% prediction accuracy [20]. Additionally, elevated levels of D-Gln and L-Gln were observed in stage IV CRC patients compared to stage I, suggesting altered amino acid metabolism in CRC progression and the potential of D-Gln as an indicator of this progression.

The present review aims to provide an integrated perspective on the role of D-amino acids in cancer, addressing their formation, analytical detection, and functional relevance across cancer types while highlighting underexplored research directions.

## 2. D-Amino Acid Synthesis

### 2.1. Endogenous Racemization

The endogenous production of D-amino acids mainly occurs via enzymatic conversion mediated by racemases, which catalyse the direct, stereochemical interconversion between L- and D-amino acids by rearranging the chiral centre of the molecule, permitting a rapid and reversible transformation, while maintaining the intracellular levels of both enantiomers.

In the human body, serine racemase was the first racemase to be identified, catalysing the conversion of L-Ser into D-Ser. D-Ser is mainly localized in the GABAergic neurons and functions as a co-agonist of NMDA ionotropic glutamate receptors, playing important roles in synaptogenesis, long-term plasticity, neuronal network activity, and cognition [21,22]. The enzyme that converts L-Asp into D-Asp, aspartate racemase, was also identified in mammals. During embryonic development, D-Asp is especially prevalent and has a role in controlling the release of neurotransmitters. It seems to also be involved in reproductive regulation since it stimulates the release of testosterone, luteinizing hormone (LH), and gonadotropin-releasing hormone (GnRH) [23]. Recently, in addition to serine and aspartate racemases, a new multifunctional enzyme with glutamate activity has been identified in mammals, expanding the enzymatic repertoire of endogenous racemases [24]. This enzyme, referred to as L-Ser/L-Thr dehydratase-like or STDHgr, is also pyridoxal-5′-phosphate-dependent, like the other known racemases. Although initially characterized as a protein of unknown function, STDHgr was later shown to catalyse the interconversion of L-Glu to D-Glu, in addition to its L-Ser and L-Thr dehydratase activity. While the physiological roles of D-Glu remain incompletely understood, its detection in various tissues suggests potential roles in amino acid homeostasis, neurotransmission, and cellular signalling [24].

### 2.2. Microbial Synthesis by the Gut Microbiota

Approximately one-third of the total D-amino acid content in the human body comes from gut microorganisms [25]. Mammalian intestines harbour a diverse gut microbiota, represented by billions of bacteria, fungi, viruses, archaea, and protozoans, many of which are capable of synthesizing D-amino acids.

Among these, bacteria are particularly important producers, presenting several other types of racemases. For example, alanine racemase, a pyridoxal phosphate-dependent enzyme, participates in peptidoglycan biosynthesis, amino acid catabolism, and spore germination regulation [26,27]. Other enzymes, such as proline racemase from *Brucella melitensis*, enhance IL-10 secretion in macrophages by activating Tpl2 and facilitating bacterial survival and persistent infection [28]. While conventional racemases are substrate-selective, recent studies have described broader-spectrum bacterial racemases, with the ability to racemize up to 10 of the 19 chiral amino acids. These enzymes represent promising targets in microbial research and bioactive compound production [29].

Beyond racemization, bacteria can use alternative enzymatic pathways for D-amino acid production, including D-amino acid aminotransferases, dehydrogenases, and oxidases. D-amino acid aminotransferases accomplish the conversion of L- to D-amino acids indirectly, prerequiring the presence of a D-amino acid (often D-Ala) as a donor of an amino group. The aminotransferases transfer the amino group to an α-ketoacid, resulting in the formation of a new D-amino acid and a corresponding α-ketoacid. D-amino acid dehydrogenases (DADs) and D-amino acid oxidases (DAAOs), although initially associated with catabolic pathways, may also support the regeneration of D-amino acids in certain bacterial species such as *Arthrobacter protophormiae* [1].

The synthesis of other D-amino acids, such as D-Tyr, D-Met, D-Try, and D-Leu, is variable, depending on individual particularities of gut flora [30]. Generally, D-amino acid production is tied to bacterial composition and competition, meaning that microbial dynamics in the gut influence the type and amount of D-amino acids available to the host. Moreover, substantial inter- and intra-species variability in D-amino acid profiles has been reported; for instance, the proportion of D-Glu among different bacterial strains ranges from approximately 4% to 49%, with *B. cereus* secreting the highest amounts, while D-Ala showed consistent levels across *Bacillus* species [30,31].

As an example, D-Trp produced by the probiotic strains LGG and *L. casei* W56 prevented the development of allergic airway inflammation via immunomodulatory effects [32]. Also, D-Ile, D-Phe, and D-Leu produced by *Staphylococcus* species activated sweet taste receptors in airways, suppressing defensin production [33]. The microbiome can also modulate the amount of D-Ser found in the blood and brain, with important neurological consequences [34]. Moreover, the gut microbiota induces the expression of DAAO in intestinal epithelial cells, including goblet cells, which converts D-amino acids into H_2_O_2_, reducing the viability of enteric pathogens like *Vibrio cholerae* [34,35]. Consequently, D-amino acids have received great attention due to their promising effects in gut health regulation and inflammatory bowel diseases [36,37,38].

### 2.3. Dietary Intake

The second most important source of endogenous D-amino acids is represented by dietary intake. Their presence in food products originates from microbial sources, such as bacteria in soil and marine ecosystems, food processing, or contamination [30,39]. D-amino acid levels are substantially increased by fermentation caused by bacteria and fungi, especially in bread, dairy products, soy sauce, or alcoholic beverages. For instance, D-Ala, D-Asp, and D-Glu are found in over half of all dairy products and fermented meals [39]. Racemization is further accelerated by processing methods such as alkali treatment or extended heating. D-amino acids are also present in ultra-processed foods due to additives like monosodium glutamate [30]. Contamination may represent another source of D-amino acids in foods due to the microbial racemases and cell wall lysis, as observed for raw milk and fruit juices during storage [40,41]. Published analyses of foods and pooled diets suggest that daily D-amino acid intake may range from 10 to 100 mg, depending on dietary composition [30].

## 3. D-Amino Acid Detection and Quantification Methods

Chiral analysis of amino acids is generally challenging and faces several major obstacles. Firstly, D-amino acids are typically present at much lower concentrations than their L-counterparts, requiring highly sensitive methods. Secondly, many amino acids lack intrinsic chromophores or fluorophores, requiring a prior derivatisation step which can introduce analytical errors and may lead to racemization under certain conditions. Moreover, due to the complexity of biological samples and the competing reactions within the sample, derivatisation cannot always be applied [7].

The state of the art in enantioselective metabolomics analyses, also covering amino acid analysis, has been described in detail in several recently published review papers, focusing on LC-MS approaches [42], indirect methods [43], or targeted analysis [44], including relevant sample preparation approaches tailored to the multitude of analytical methods.

The analytical methods used to identify and/or quantify D-amino acids in human samples include separative and biosensor-based techniques.

### 3.1. Separative Methods

The vast majority of separative methods used for D-AA analysis are based on liquid chromatography and capillary electrophoresis techniques, coupled with diverse detectors. High-performance liquid chromatography (HPLC), with its ongoing advancements, remains the most important analytical technique for separating amino acid enantiomers. Their separation can be achieved directly (based on enantiospecific interactions with a stationary phase) or indirectly (based on pre-column derivatization, followed by reversed phase (RP) chromatography).

The earliest methods implied an initial derivatization step using chiral reagents, such as o-phthaldialdehyde (OPA) in combination with chiral thiol compounds, to convert amino acids into diastereomeric isoindole derivatives. These derivatives were highly fluorescent and could be determined by a sensitive fluorescence detector. Other commonly used chiral derivatization reagents include Marfey’s reagent, or 1-fluoro-2,4-dinitrophenyl-5-L-alanine amide (FDAA), (+)- or (−)-1-(9-fluorenyl)ethyl chloroformate ((+) or (-)-FLEC)), or (S)-*N*-(4-Nitrophenoxycarbonyl)-L-phenylalanine-2-methoxyethyl ester ((S)-NIFE). Subsequently, the diastereomers are analysed using conventional achiral stationary phases [45]. Such a one-dimensional HPLC method was employed to measure five D-amino acids (D-Ser, D-Ala, D-Pro, D-Asp, and D-Glu) in human gastric juice and cultures of *H. pylori* cells using an FDAA derivatization reagent and UV detection [46]. Similarly, enantioselective analysis of Thr, Ala, Tyr, Val, and Phe in patients with hepatocellular carcinoma was performed using pre-column derivatization with OPA and *N*-isobutyryl-L-cysteine (NAC), followed by fluorescence detection (FLD) [47]. Compared to HPLC, ultra-high-performance liquid chromatography (UHPLC) enabled shorter run times due to higher pressure capabilities. Moreover, the UHPLC columns have smaller particle sizes, enhancing separation efficiency. While UV or FLD detectors require derivatization to improve detection, the transition to mass spectrometry (MS) constituted another notable improvement, as it allowed for direct analysis of amino acids (or required minimal derivatization steps) and provided superior sensitivity and specificity. Also, tandem mass spectrometry (MS/MS) detection minimized background noise and matrix effects, leading to more accurate quantification of D-amino acids. Such sensitive UHPLC-MS methods (LODs in the range of pM to nM) have been developed for D-amino acid analysis in human serum, plasma, urine, and mouse gut [48] or rat brain [49]. Further advances have concerned derivatization agents, particularly the introduction of bromine or chlorine-labelled probes. These offer high sensitivity due to their specific characteristics (the presence of ^79^Br/^81^Br or ^35^Cl/^37^Cl isotopes, respectively) and facilitate analyte identification, improving quantification accuracy [43]. Such derivatization agents include 1-benzoyl-pyrrolidine-2-carboxylic acid 5-chloro-2-formyl-phenyl ester probe with the D-steric configuration (D-BPCl), used for the HPLC-MS/MS profiling of intra- and extracellular L- and D-amino acids in colorectal cells and intestinal epithelial cells [50] or for the very sensitive (LODs in low nM range) HPLC-MS/MS profiling of 14 chiral amino-containing metabolites in urine samples of gastric patients and healthy controls [51].

The separation of D-amino acids can be also achieved by using chiral stationary phases. Often, this implies a two-dimensional liquid chromatography (2D-LC) approach, first using an RP column for the first dimension and an enantioselective column for the second dimension. By combining two complementary separation mechanisms, this approach improves the detection of low-abundance D-amino acids in complex biological samples. Numerous 2D-LC methods have been developed for the precise analysis of D-amino acids in biological matrices, using either FLD [52,53] or MS detection [54,55,56]. In the context of cancer research, Nakade et al. [57] used a 2D-LC approach to measure L- and D-amino acids (Ser, Ala, Asn, and Pro) in resected brain tissues, blood, and urine from patients with primary gliomas. Tsugaru et al. [58] assessed the role of D-Ser, D-Asn, D-Ala, and D-Proas diagnostic or predictive biomarkers in gastric cancer patients. Current and advanced LC/MS techniques, including sample preparation methods for chiral molecules as disease biomarkers, can be found in a recently published comprehensive review [59].

Far fewer gas chromatographic (GC) methods have been developed for D-amino acid analysis due to their low volatility and the need for extensive derivatization. One of the first GC-MS methods for enantioseparation was developed in 1965, using artificial mixtures of D- and L-amino acids [60], but application on biological samples emerged only later [61,62,63]. However, to our knowledge, no GC methods have been applied in the analysis of D-amino acids as potential biomarkers for cancer onset or progression.

Capillary electrophoresis (CE) was also employed in the chiral separation of amino acids due to small sample volumes (100 nL–1 mL) and relatively short separation times [8]. However, most studies focused on neurological disorders [64,65,66]. Despite its advantages, the application of CE for chiral amino acid analysis in oncological research remains relatively unexplored.

### 3.2. Biosensors

All of the above methods have clear advantages in terms of sensitivity and selectivity, especially when coupled with an MS detector. However, they are relatively expensive techniques, require specialized training particularly for method development and data analysis, and cannot be used in online applications. These drawbacks are overcome by biosensors, which are more cost-effective, easy to use, and can be integrated into portable devices [67]. A comprehensive review on the detection methods of D-amino acids using biosensors and their applications for human health has been published by Rosini et al. [67]. Over time, different biosensors have been developed, based on the physical change generated by the biological reaction: calorimetric, potentiometric, amperometric, optical, or piezoelectric biosensors [67]. Most methods use the flavoenzyme DAAO, which catalyses the oxygen-dependent deamination of D-amino acids into their corresponding α-ketoacids, ammonia, and hydrogen peroxide [67].

For measuring D-amino acids, several sensors and biosensors have been developed, particularly for the early diagnosis of gastric cancer, including luminescent DNA/silver nanocluster-based biosensing systems [68], stochastic sensors for tryptophan enantioanalysis [69], or sensors that use nanozymes with peroxidase-like activity (POD-mimic) such as carbon quantum dots in nitrogen-doped carbon [70] or copper(I)-based metal–organic complexes [71] with colorimetric detection or electrochemiluminescence [72]. Recently, a novel method used a nanoreactor based on a mesoporous hydrogen-bonded organic framework (MHOF) with visual detection based on chromogenic reactions for the enantiospecific profiling of D-amino acids in the saliva and serum of gastric cancer patients. The use of the MHOF-based nanoreactor led to improved enzyme stability and reduced detection times [73].

**Figure 1:**
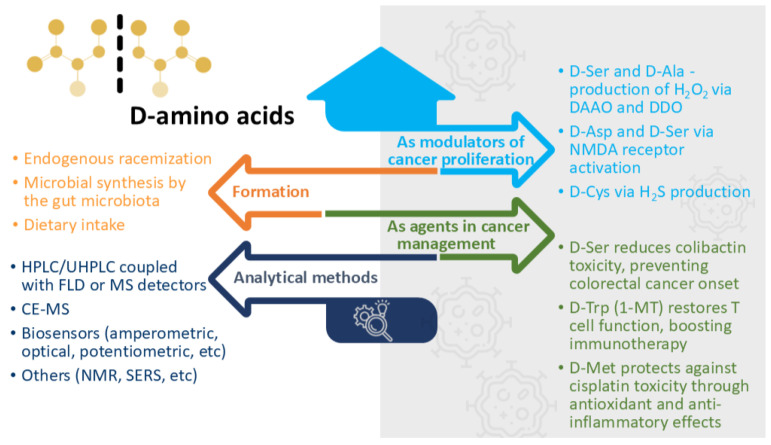
Formation routes, analytical methods, and the involvement of D-amino acids in the modulation and management of various cancers (template by PresentationGO, www.presentationgo.com, accessed on 20 March 2025, adapted). Abbreviations: 1-MT, 1-Methyltryptophan; DAAO, D-amino acid oxidase; DDO, D-aspartate oxidase; HPLC, high-performance liquid chromatography; FLD, fluorescence detection; MS, mass spectrometry; NMR, nuclear magnetic resonance; SERS, surface-enhanced Raman spectroscopy; UHPLC, ultra-high-performance liquid chromatography.

Apart from the methods described above, the analysis of D-amino acids has been conducted using less common techniques, such as nuclear magnetic resonance [74], surface plasmon resonance [75], or Raman spectroscopy [76,77], as presented in Figure 1.

As seen, each method has its strengths and limitations. Therefore, the integration of biosensors with traditional separative methods could result in more comprehensive, accurate, and cost-effective solutions for D-amino acid analysis.

### 3.3. Analytical Perspectives

The efforts of the scientific community continue to focus on overcoming the shortcomings encountered in the enantioselective analysis of amino acids, such as limited throughput, tedious sample preparation protocols, or limited sensitivity or specificity. For example, the study published recently by Jaag et al. [78] describes a high-throughput liquid chromatography with ion mobility–mass spectrometry (LC-IM-MS) approach for D-amino acid analysis. The baseline separation of 17 pairs of proteinogenic amino acids was achieved in less than 3 min. This remarkable separation was based on pre-column derivatization of amino acids with 6-aminoquinolyl-*N*-hydroxysuccinimidyl carbamate (AQC), followed by LC separation on a tandem column setup based on weak anion exchange and zwitterionic-type quinine carbamate selectors. Moreover, ion mobility spectrometry enabled the confirmation of Thr isomers but could not differentiate among Leu, Ile, and allo-Ile enantiomers. Chiral discrimination of amino acid enantiomers by trapped ion mobility spectrometry has been previously reported by Pérez-Míguez et al. [79], with 17 (+)-FLEC derivatives being separated in around 3 min. Even though not applied on biological samples, these studies provide strong evidence regarding the usefulness of ion mobility spectrometry applications for chiral analysis of amino acids. Therefore, ion mobility spectrometry, with or without prior LC separation, may eventually play a pivotal role in achieving the required throughput, selectivity, and specificity of standardized chiral assays useful for clinical applications. Moreover, applications such as real-time D-amino acid imaging might be achievable once IM technology is able to provide sufficient separation power to resolve ions with close collisional cross-section values.

Liquid chromatography remains the most used technique for enantioselective amino acid profiling, offering alternatives tailored to different needs, such as fast separation for high-throughput profiling [78] to high sensitivity for targeted analyses [80] or robust approaches for routine analysis. Nevertheless, capillary electrophoresis can be an alternative in some cases, mostly in relatively clean matrices (such as CSF or urine), providing similar performance at a fraction of the cost [81]. In addition, biosensors could complement these approaches by offering the possibility of targeted measurements in non-laboratory environments.

## 4. D-Amino Acids in Tumours

The presence of D-amino acids in tumour proteins was first reported by Kögl and Erxleben in 1939 [82]. According to their results, the development and progression of tumour cells depended on D-amino acid formation, particularly D-Glu. For decades, their observations were questioned, with several studies either supporting or contradicting their results [83,84,85]. A more thorough examination of these conflicting findings was made possible by the methodological developments in MS detection and ^14^C-labelled procedures in the second half of the 20th century [86]. Fisher et al. [87] claimed that D-amino acids were not universally present in all tumour tissues and were not considered essential for cancer development. The same group’s later research concluded that there was no significant difference in D-Asp and D-Glu levels between tumour and normal tissues [88]. However, recent investigations conducted using advanced enantioseparation techniques have identified altered D-amino acid levels in certain cancer types, indicating possible roles in tumour growth, therapy resistance, and biomarker identification [20,70,89,90]. Table 1 summarizes the types of cancer and the biological samples in which D-amino acids have been investigated and the main outcomes.

As shown in Table 1, D-amino acid profiles differ between cancer patients and healthy controls. As these findings are based on observational data from biological samples, they primarily indicate correlation rather than causation. It remains debatable whether the observed D-amino acid changes contribute to tumour development, result from altered tumour metabolism, or reflect systemic responses. A possible explanation was offered by Du et al. [90], which demonstrated that, in MCF-7 breast cancer cells, intracellular L-Asn and D-Asn exchanges with extracellular amino acids, such as L-Ser, L-Arg, and L-His, via antiport transporters. Therefore, L and D-Asn may serve as exchange currency for the uptake of essential amino acids and/or low-abundance nonessential amino acids that are required by cancer cells during proliferation. Earlies studies have demonstrated that the maintenance of intracellular Asn levels is critical for cancer cell growth [93]. When selected D-amino acids were exogenously administrated, they generally impaired cell proliferation through the mechanisms detailed in Section 5.1 and Section 5.2.

However, when evaluating the translational potential of D-amino acids for diagnostics or therapeutic purposes, several variables should be considered. Inter-patient variability may affect both the synthesis and systemic levels of D-amino acids, driven by differences in gut microbiota composition, as described above, dietary habits, and metabolic particularities. Additionally, confounding factors such as co-morbidities, immune status, tumour heterogenicity, the microenvironmental context, and enzymatic activity (e.g., racemase, DAAO status) may influence D-amino acid levels and biological effects. Furthermore, differences in analytical approaches and sample types across studies may further complicate D-amino acid interpretation in cancers and should be carefully considered.

## 5. Implications of D-Amino Acids in Cancer

### 5.1. The Manipulation of D-Amino Acid Levels as a Tool for Regulating Cell Proliferation

Cancer cells require adequate levels of proteinogenic amino acids in order to proliferate and survive. Temporarily replacing a normal diet with an artificial one that selectively modifies amino acid levels or manipulating amino acid levels in the tumour microenvironment could potentially hinder cancer cell growth and survival [94,95]. In this light, several studies have investigated the impact of exogenous D-amino acid supplementation on tumour progression.

The influence of amino acids on the survival of CHO and HeLa cell lines was assessed using colony formation assays, with cells exposed to millimolar concentrations of D- and L-amino acids [96]. Interestingly, the proliferation of both cell lines was impaired by both D- and L-forms of Lys, Arg, Phe, Trp, Asp, and Glu, with D-enantiomers exerting a significantly stronger inhibitory effect on cell survival. According to the study, the cytotoxic effect was, at least partially, due to the oxidation of D- and L-amino acids, leading to H_2_O_2_ production, which induced lipid peroxidation and oxidative stress. On the other hand, D-Cys supplementation on the Hepa 1–6 mouse hepatoma-derived cell line reduced lipid peroxide accumulation and maintained intracellular L-Cys and glutathione (GSH) levels. Also, D-Cys temporarily protected cells from ferroptosis by supporting L-Cys uptake and GSH synthesis, though it did not serve as a direct GSH source [97]. In fact, a more recent study demonstrated that different D-amino acids use distinct mechanisms to trigger inflammatory responses and subsequent cell death [98]. Exposure of HepG2 liver cancer cells to D-Ser caused downregulation of D-amino oxidase mRNA expression and decreased H_2_O_2_ production, leading to concentration-dependent mitochondrial membrane depolarization and finally apoptosis. This caspase-independent apoptosis might be mediated via the GCN2 pathway, a cellular sensor of amino acid starvation.

In contrast, D-Ala-treated cells upregulated both D-amino oxidase mRNA and protein expression at low concentrations, induced H_2_O_2_ production, and triggered inflammatory markers, without causing significant apoptosis. Notably, both D-Ser and D-Ala upregulated NF-κB activation and increased TNF-α pro-inflammatory cytokine and IL-8 chemokine secretion [98].

Another D-amino acid, D-2-Hydroxyglutarate (D-2HG), was shown to interfere with anti-tumour immunity. D-2HG, an oncometabolite produced by IDH1/2-mutant tumours, was shown to directly impair CD8^+^ T cell anti-tumour functions. The direct mechanism involved the inhibition of lactate dehydrogenase, the disruption of glycolytic metabolism, and a decrease in the NAD^+^/NADH ratio. This led to reduced glycolytic flux, mitochondrial hyperpolarization, and increased reactive oxygen species production. As in IDH-mutant gliomas, D-2HG accumulates and correlates with lower CD8^+^ T cell infiltration and activity; targeting D-2HG might improve T cell-mediated anti-tumour immunity [99]. This mechanism contributes to immune evasion in IDH-mutant tumours, highlighting D-2HG as a metabolite that actively suppresses anti-tumour immunity.

In an animal study, specific D-amino acids were infused into AH 109A hepatoma-bearing rats via total parenteral nutrition. The group receiving D-Val experienced a notable reduction in tumour growth, as well as a decrease in DNA and protein content in the tumour tissue without negatively impacting the host’s nutritional status. Also, the D-Leu and D-Met groups showed improved liver protein and DNA content, suggesting improved nutritional status. In addition, D-Met prevented transferrin decrease and improved survival time, suggesting that this amino acid could counteract malnutrition in tumour-bearing rats [100]. The same favourable profile of D-Met was observed in vitro, with D-Met-supplemented medium inhibiting the growth of AH109A hepatoma cells, most likely due to suppressed protein synthesis in tumour cells [101].

When MCF-7 breast cancer cells were exposed to high concentrations of D-Leu (50 µM for 24 h), a similar growth inhibitory effect was observed. D-Leu reduced the uptake of essential amino acids via system L, an effect that could not be attributed to osmolarity changes, as demonstrated by control experiments using sucrose and urea [102]. However, not only D-amino acid supplementation can impact breast cancer proliferation. When the same cell line was exposed to high concentrations of glucose (25 mM), a clear proliferative effect was observed, particularly after 72 h. This cell growth was accompanied by higher levels of free L-amino acids, especially L-Gln (681 nmol/10^6^ cells in high glucose versus 377 nmol/10^6^ cells in normal glucose after 24 h), Gly, and L-Glu. In contrast, intracellular L-amino acid trends remained similar, regardless of glucose concentration. Similarly, while most D-amino acid levels remained unchanged, D-Thr and D-Ser showed increased levels in high-glucose conditions, especially after 24 h [90].

A recent study demonstrated that supplementation of cell culture medium with high concentrations of mixed D-amino acids caused a significant decrease (from ~60% to 26.6%) in HCT116 colorectal cancer cells. Moreover, an increased addition of D-amino acids in HCT116 cells led to a significant reduction in L-AABA, L-Asn, L-Thr, and L-Met levels, while L-Val levels increased. In contrast, D-amino acid supplementation had no significant effect on the growth and amino acid profile of NCM460 normal intestinal epithelial cells. Notably, L-Ala showed opposite trends in the two cell lines, suggesting distinct metabolic responses between the tumour and normal cells [50].

The modulation of amino acid levels, either through dietary interventions as seen in animal studies or by altering the tumour microenvironment in vitro, emerges as a potential strategy to suppress cancer growth. In general, D-enantiomers exhibit stronger inhibitory effects compared to their L-counterparts. Moreover, the differential metabolic responses between normal and cancerous cells to D-amino acid manipulation suggest that this is a promising tool for targeted therapeutic approaches.

### 5.2. D-Amino Acids as Modulators of Cancer Proliferation Pathways

As presented above, D-amino acid metabolism can produce H_2_O_2_ via DAAO and D-aspartate oxidase (DDO) [96,98]. At physiological levels, H_2_O_2_ acts as a secondary messenger involved in cell signalling, including proliferation, differentiation, and migration. However, at elevated levels (>100 nM), H_2_O_2_ can act as a reactive oxygen species, inducing oxidative stress, mitochondrial dysfunction, and activation of inflammatory cytokines, ultimately contributing to cell injury and tissue and organ decline [98,103]. In vitro chemogenetic models using DAAO have shown that H_2_O_2_ induces rapid and localized oxidative stress, modulating redox-sensitive pathways, such as phosphorylation cascades and transcription factor activation (such as Nrf2, NF-κB). Sustained or excessive intracellular H_2_O_2_ can overwhelm antioxidant defences, leading to protein, lipid, and DNA oxidative damage. Ultimately, this oxidative distress triggers cell death pathways, including apoptosis or necrosis, depending on the intensity of the redox imbalance [103]. As validation of these observations, when PEGylated DAAO was systemically administered in combination with D-Pro as substrate, it generated cytotoxic levels of H_2_O_2_ selectively at the tumour site, leading to significant tumour regression without harming normal tissues [104]. However, an experimental study showed that the oxidative damage resulting from H_2_O_2_ production from certain D-amino acids (D-Ala, D-Pro, and D-Lys) was not the only mechanism contributing to their toxicity [105]. Instead, additional H_2_O_2_-independent pathways could contribute to the apoptosis-mediated pathways.

One of the other implications of D-amino acids in cancer proliferation was attributed to their capacity to modulate NMDA receptors. When overactivated, these receptors mediate an excessive increase in Ca^2+^, leading to excitotoxic cell death (cell death resulting from the toxic actions of excitatory amino acids, primarily glutamate), as seen in Alzheimer’s or Huntington’s disease. Interestingly, NMDA receptors can also be involved in cell cycle alterations and aberrant cell growth, as seen in glioblastoma and different cancer types [106,107,108,109]. In pancreatic neuroendocrine tumorigenesis, NMDA activation was shown to trigger different signalling pathways such as mitogen-activated protein kinase (MEK–MAPK), which promotes cell proliferation, and survival or calcium/calmodulin-dependent protein kinase IV (CaMK-IV), which enhances cell invasion [110].

Four D-amino acids modulate NMDA receptors, acting either as an agonist at the glutamate site (D-Asp and D-Glu) or the glycine binding site (D-Ala and D-Ser). Other D-enantiomers, such as D-Ile, D-Leu, D-Phe, D-Thr, and D-Tyr, are considered neuroactive D-isomers without directly interacting with NMDA receptors [111].

In MCF-7 breast cancer cells, D-Asp and D-Ser showed significantly elevated levels compared to non-tumorigenic MCF-10A cells (3 to 22 times higher). Given that functional NMDA receptors are expressed by both cell lines, these D-amino acids could induce NMDA receptor activation [90,109]. The same conclusion was drawn for Hs 895.T skin cancer cells and their normal counterpart, Hs 895.Sk skin cells, both expressing NMDA receptors [112]. Moreover, when skin cancer cells were treated with NMDA receptor blockers (MK-801 or memantine), the addition of D-Ser, D-Asp, or D-Ala reversed the antiproliferative effect, restoring cell growth. This observation suggests that cancer cells may rely on NMDA receptor activation for survival and progression. Additionally, the authors noted that NMDA receptor inhibition can disrupt the extracellular signal-regulated kinase (ERK) pathway, which is generally linked to cell proliferation, cell survival, and cell migration. They proposed that a similar mechanism may underlie the observed growth inhibition in their skin cancer model, although it was not directly tested in the study [112].

Another modulating mechanism of D-amino acids on cancer proliferation concerns the production of H_2_S through D-Cys. D-Cys is first oxidized by DAAO to 3-mercaptopyruvate, NH_3_, and H_2_O_2_. Subsequently, 3-mercaptopyruvate is metabolized by 3-mercaptopyruvate sulphur transferase (3-MST), finally leading to H_2_S production [113]. The formation of H_2_S from D-Cys via DAAO has been demonstrated in the rat jejunal mucosa [114] and mice [115]. H_2_S, considered a gaseous transmitter, generally exerts a biphasic effect in tumours. At endogenous levels or low exogenous concentrations, it promotes cancer cell growth by increasing angiogenesis, mitochondrial bioenergetics, and antioxidant defence. In contrast, higher concentrations of H_2_S exert anti-cancer effects by inducing apoptosis, DNA damage, and cell cycle inhibition [116]. Numerous studies have demonstrated that H_2_S interferes with multiple signalling pathways such as PI3K/Akt/mTOR and MAPK, thereby supporting the development of various cancer types [113,117,118,119].

In a study designed to evaluate the concentration and effects of H_2_S generated from D-Cys via DAAO in the gastric mucosa of mice, the results showed that pretreatment with D-Cys significantly reduced ethanol-induced gastric lesions, including haemorrhagic damage, oedema, and epithelial loss. The protective effect of D-Cys was clearly mediated by H_2_S production, as inhibition of DAAO with indole-2-carboxylate reversed these effects. Additionally, D-Cys exerted an antioxidant role, reducing malondialdehyde levels while maintaining the levels of reduced glutathione [120]. Also, H_2_S derived from D-Cys enhanced chaperone-mediated autophagy activity via Nrf2 activation, suggesting a potential therapeutic strategy for spinocerebellar ataxia [121]. Additionally, D-Cys has been shown to protect cerebellar neurons from oxidative stress and to attenuate ischemia–reperfusion injury in the kidney [122]. Novel mechanisms suggest that endogenous D-Cys acts on protein kinase B (AKT) signalling and binds to Myristoylated Alanine Rich C Kinase Substrate (MARCKS), altering phosphorylation at Ser 159/163 and its translocation from the membrane [123]. While these observations were made in the context of psychiatric disorders, their potential relevance to cancer deserves further investigation.

Synergistic effects were observed when D-amino acids were combined with conventional chemotherapeutic agents. Co-administration of D-Lys and doxorubicin significantly enhanced (*p* < 0.05) cytotoxicity in MCF-7 breast cancer cells [124]. More studies have focused on incorporating D-amino acids into anti-tumour peptides, yielding promising results. As an example, the D-amino acid variant (9D-RDP215) of the lactoferricin-derived peptide displayed greater structural stability, higher membrane interaction, and enhanced cytotoxicity in both 2D and 3D glioblastoma models compared to its L-analogue, thus indicating a therapeutic potential for brain tumours [125]. Similar enhanced biostability and inhibitory activities have been reported for D-amino acid peptides in lung [125], colon and breast [126], prostate [127], and kidney cancer cells [89]. Several new synthetised D-amino-acid-containing peptides with possible oncological applications have already been patented [128,129].

### 5.3. Specific D-Amino Acids as Functional Agents in Cancer Management

D-amino acids interfere in cancer management through various mechanisms, such as preventing tumour onset, strengthening immune defence, or mitigating chemotherapy side effects.

Hallam et al. [130] established a close connection between the development of colorectal cancer and colibactin, a genotoxic compound produced by *pks*+ *Escherichia coli.* Colibactin production appears to induce DNA double-strand breaks, DNA crosslinks, and chromosomal instability, which have been linked to colon cancer development. D-Ser was found to significantly inhibit colibactin production by downregulating *clb* genes in *pks*+ *E. coli.* This triggered a reduced crosslinking formation and less DNA damage in HeLa cell infection. Moreover, exposure of HeLa cells to D-Ser reduced the cellular senescence induced by colibactin. These results support the fact that D-Ser could efficiently prevent colorectal cancer onset.

Another D-enantiomer, D-Trp, has been investigated in its prodrug form, 1-methyl-D-tryptophan (1-MT). 1-MT, also known as Indoximod, is an inhibitor of indoleamine 2,3-dioxygenase (IDO), an enzyme that inhibits T cell anti-tumour immunity [131]. High IDO expression in solid tumours has been linked to poor prognosis, shorter overall survival, and chemoresistance [132,133]. Targeting IDO represents a therapeutic option in cancer immunotherapy, and several phase I and II studies have explored the role of 1-MT, alone or in combination with docetaxel, sipuleucel-T, or ipilimumab, in breast, prostate, pancreas, and other cancers [134].

There is consistent evidence on the role of D-Met as a protective agent against the adverse effects of cisplatin. Cisplatin, a platinum-based chemotherapy drug, is used in the treatment of various cancers, including carcinomas, germ cell tumours, lymphomas, and sarcomas [135]. Most patients receiving cisplatin typically experience dose-limiting adverse effects such as nephrotoxicity, peripheral neuropathy, nausea, and vomiting [135]. Specifically, D-Met has been shown to provide protection against cisplatin-induced ototoxicity [136,137] and vestibulotoxicity [138], alleviate anorexia and dyspepsia syndrome [139], reduce nephrotoxicity [140], and ameliorate muscle atrophy associated with cisplatin treatment [141]. The otoprotective effects of oral D-Met were validated in a phase 2 randomized clinical trial, where the D-Met group (n = 14) showed no significant shifts at any tested frequency, while the placebo group (n = 13) exhibited significant shifts at 10, 11.2, and 12.5 kHz [137]. The protective effects of D-Met against oto- and nephrotoxicity were attributed to its antioxidant effects, preserving the levels of endogenous antioxidants such as superoxide dismutase, catalase, and glutathione, while preventing the accumulation of malondialdehyde. Additionally, this antioxidant activity was frequently accompanied by an anti-inflammatory effect. For example, in cisplatin-induced mucositis, D-Met restored the gut microbiota structure and reduced intestinal inflammation [142]. The same antioxidant and anti-inflammatory effects of D-Met have been displayed for methotrexate-induced nephrotoxicity in rats [143].

So far, only a few D-amino acids have been included in clinical evaluation. These clinical trials were designed mainly for neurological and oncological conditions. D-Ser has been studied in schizophrenia, Parkinson’s disease, and cognitive decline, showing improvements in symptom severity and cognitive function at doses of 30 mg/kg/day over 6 weeks. Similar results were obtained for D-Ala in schizophrenia [18,144]. In oncological-related trails, only D-Met has been clinically evaluated so far, showing protective effects against cisplatin- and noise-induced hearing loss, as presented above [137]. To our knowledge, no clinical trials have yet demonstrated that D-amino acid manipulation alone or in combination with chemotherapy yields therapeutic efficacy in terms of tumour size reduction or direct inhibition of cancer cell proliferation.

A general presentation of D-amino acids, from formation pathways to analytical methods used in their detection and proposed mechanisms of action, is presented in Figure 1.

## 6. Future Research Directions

**Figure 2:**
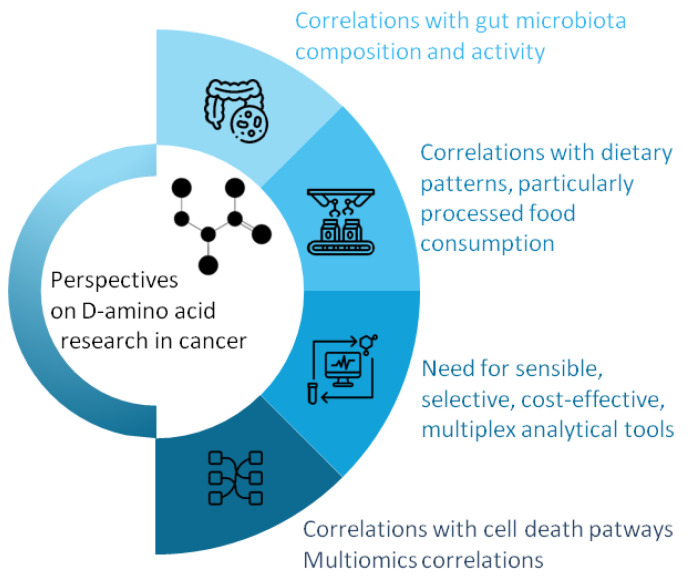
Emerging research directions of D-amino acids in cancer (template by PresentationGO—www.presentationgo.com, accessed on 21 March 2025, adapted).

Based on the current knowledge, we have identified several possible research directions regarding the impact of D-amino acids on cancer development and progression, as presented in Figure 2.

Microbially derived D-amino acids are believed to represent approximately one-third of the overall D-amino acid content in the human body [25]. Increasing evidence sustains that the gut microbiome and its metabolites can act as cancer promotors or inhibitors, trigger cancer immune responses, or alter the efficiency of chemotherapeutic drugs [145,146,147]. As D-amino acids are essentially required for the peptidoglycan walls of bacteria, their elevated presence in several malignancies, such as gastric cancer (as seen in saliva samples of gastric cancer patients [68,70,73,76]), warrants further investigation. Further studies should correlate the D-amino acid profiles (especially D-Pro and D-Ala for gastric cancers) with gut microbiome composition and activity to seek possible links or novel mechanistic insights.

Dietary intake also contributes to D-amino acid accumulation. During food processing, the D-amino acid concentrations in foods and beverages increase significantly [30]. The consumption of processed and ultra-processed products has constantly increased, causing elevated levels of D-amino acids and chemical alterations within the gastrointestinal tract [30]. Moreover, an increasing amount of data indicate a strong correlation between the consumption of ultra-processed foods and the risk of developing several cancer types [148,149]. In this context, future studies analysing D-amino acids should consider assessing the relationship between D-amino acid levels and dietary patterns, with particular attention paid to processed and ultra-processed food consumption.

The analytical techniques used to investigate D-amino acids often lack selectivity and/or sensibility and are typically expensive, which limits their use in clinical applications. To overcome these drawbacks, an ideal tool for detecting D-amino acids in oncology should have ultra-high sensitivity and specificity and be capable of non-invasive, real-time, and cost-efficient analysis from various biological samples like blood, urine, saliva, or tissue biopsies. Miniaturization, portability, and smart integration for real-time monitoring should also be considered. Ideally, this tool should be capable of detecting trace levels of D-amino acids in the presence of large L-amino acid quantities. As a promising example, recently developed H_2_O_2_-regulated split-type electrochemiluminescence with a MnO_2_/AuNC-based nanoswitch platform demonstrated an extremely remarkable sensitivity (with a limit of detection of 2.2 × 10^−11^ M) and high selectivity for D-Ala detection in saliva samples [150]. Novel enzyme variants that specifically recognize individual D-amino acids can be developed and used as bioreceptors in detection systems [67]. Moreover, simultaneous analysis of multiple D-amino acids and clinical variables could reveal distinct signatures associated with cancer type, stage, or treatment response. For instance, such a logistical regression model was established by Huang et al. [51] for the diagnosis of gastric cancer. The model included D-Ile, D-Ser, β-(pyrazol-1-yl)-L-alanine, and age and achieved an average prediction correctness of 88.9% in the validation set. Similarly, a panel composed of age, L-Ala, D-Ala, D-Gln, D-Ser, and D-AABA discriminated the colorectal patients from healthy controls with 96.7% prediction accuracy, while the four D-amino acids alone reached 95.2% prediction accuracy.

Also, a deeper understanding of the biochemical pathways of D-amino acids in cancer onset, progression, and chemoresistance is needed. For this purpose, future studies could investigate whether imbalances in D/L-amino acid ratios reflect tumour hypoxia, oxidative stress, or metabolic rewiring during cancer transformation. Moreover, D-amino acid metabolomics should integrate with genomics, proteomics, or microbiomics. As datasets in multiomics are often challenging to correlate, AI algorithms could help identify biomarker patterns based on individual D-amino acid signatures.

## 7. Conclusions

Over the last ten years, the interest in D-amino acids as possible biomarkers with clinical applications has increased [10,50,71,151]. Initially considered irrelevant for eukaryotic cells, D-amino acids are now recognized as important players in neurotransmission, immune regulation, and even cell proliferation.

Endogenous D-amino acid levels found in the saliva, whole-blood samples, serum, and gastric juice of cancer patients continue to have debatable roles [151], with some studies reporting no significant differences in D-amino acid ratios between cancerous and non-cancerous groups [46], while others construct highly sensible and specific diagnostic biomarker models based on D-amino acids [20]. These discrepancies may be due to differences in analytical methodologies, sample types and sample preparation, patient heterogeneity, tumour type and stage, or microbiome composition.

Exogenous manipulation of D-amino acid levels has generally demonstrated selective cytotoxicity toward cancer cells while sparing normal cells, often exerting stronger inhibitory effects than their L counterparts. So far, several mechanisms have been proposed for explaining the role of endogenous D-amino acids in cancer proliferation: the production of H_2_O_2_, the modulation of NMDA receptor activity, and the production of H_2_S via D-Cys metabolism, causing antioxidant and anti-inflammatory effects. In addition, specific D-amino acids such as D-Ser, D-Trp, and D-Met have demonstrated the potential to prevent tumour onset, enhance immune response, or reduce chemotherapy-induced side effects, respectively.

Besides standardization of analytical procedures, well-controlled, large clinical studies are essential to validate their relevance and utility as reliable biomarkers. Moreover, a better understanding of the interplay between D-amino acid metabolism and cellular mechanisms, processed food consumption, and the gut microbiome, along with advances in enantioseparation and detection, could provide new perspectives on their role in cancer diagnosis and therapy.

## Figures and Tables

**Table 1:** A global overview of research studies investigating D-amino acids in human cancer cells, tissues, or biological fluids, including the analytical methods and the main outcomes.

Sample Type	Analytical Method	D-Amino Acids Studied	Main Outcomes	Ref.
*Gastric cancer (GC)*				
Gastric juice from GC patients and controls (+/− infection with *H. pylori*)	Derivatization followed by RP-HPLC-UV separation	D-Ser, D-Ala, D-Pro, D-Asp, D-Glu	No significant correlation between overall D-amino acid ratios and the presence of GC D-Ala levels were notably higher in patients with both GC and *H. pylori* infection compared to other groups	[46]
Urine from GC patients and controls	RP-HPLC-MS/MS in combination with chiral chlorine-labelled probes	D-Asn, D-Cit, D-Gln, D-Thr, D-Ser, D-Ala, D-Tyr, D- Val, D-Met, D-Trp, D-Leu, D-Ile, D-Phe, D-AABA	18 differential variables (including 7 D-amino acids) were assumed to have diagnostic value for GC A diagnostic regression model including D-Ile, D-Ser, β-(pyrazol-1-yl)-L-alanine, and age was validated with 88.9% prediction accuracy	[51]
Whole-blood samples from GC patients and controls	Enantioselective stochastic sensors	D-Glu	Both D- and L-Glu were found in GC patients, while only L-Glu was detected in controls D-Glu could serve as an early diagnostic biomarker for GC	[91]
	Enantioselective stochastic sensors	D-Try	Both D- and L-Try were found in GC patients, while only L-Try was detected in controls The levels of L-Try in controls were higher than in GC patients L-Tyr could serve as a biomarker for GC	[69]
Plasma from GC patients before treatment and healthy controls Plasma from advanced GC patients before nivolumab	2D HPLC-MS/MS	D-Ser, D-Asn, D-Ala, D-Pro	D-amino acid levels were notably higher in GC patients, even in stage 1 patients D-Ser levels increased with disease progression High D-Ser levels were associated with poor efficacy and prognosis in patients treated with nivolumab D-amino acids could serve as a novel biomarker of both GC detection and the efficacy of nivolumab	[58]
Saliva from GC patients and controls	Non-invasive biosensing system with luminescent DNA/silver nanoclusters	D-Ala, D-Pro	D-amino acid levels were significantly higher in GC patients, making them potential non-invasive biomarkers	[68]
	Carbon dots confined in *N*-doped carbon nanozyme with peroxidase-like activity (with colorimetric detection)	D-Pro, D-Ala	D-Pro + D-Ala levels were higher in GC patients compared to controls D-Pro + D-Ala levels could be a useful indicator for early GC diagnosis	[70]
	CuX-trithiocyanuric acid complexes with peroxidase-like activity (with colorimetric detection)	D-Pro, D-Ala	L-Pro concentrations in GC patient samples were approximately 16–45 times higher than in healthy controls (255–448 μM vs. 10–16 μM) The method could be applied for the preliminary screening and diagnosis of GC	[71]
	SERS coupled with a DAAO-mediated cascade reaction	D-Pro, D-Ala	The total levels of D-Pro and D-Ala in gastric cancer samples were higher than those of healthy controls	[76]
	Biocatalytic nanoreactor based on mesoporous hydrogen-bonded organic framework	D-Phe, D-Ser, D-Ala, D-His, D-Arg, D-Orn, D-Asp	D-amino acid levels were low in the serum samples of both GC patients and healthy individuals The concentrations of D-Ala and D-Pro were significantly higher in the saliva of GC patients compared to healthy individuals	[73]
*Hepatic cancer (HC)*				
Serum from HC patients and healthy controls	HPLC–MS/MS	D-Arg, D-Ile, D-Asp, D-Trp, D-Ala, D-Glu, D-Tyr, D-His, D-Asn, D-Met, D-Ser, D-Gln, D-Cys, D-Val, D-Leu, D-Phe, D-Thr	D-Glu, D-Gln, D-Ile, D-Ala, D-Met, D-Thr, and several L-amino acids showed significantly reduced concentrations in HC patients compared to healthy individuals D-Glu and D-Gln showed the most significant reductions in HC patients (fold change > 1.5), serving as potential biomarkers for HC diagnosis and research	[92]
Plasma from HC patients and healthy controls	HPLC-FLD	D-Thr, D-Ala, D-Tyr, D-Val, total Met	D/L-amino acid patterns differed significantly between HC patients and healthy individuals (particularly L-Thr, D-Thr, L-Ala, and D-Ala levels)	[47]
*Colorectal cancer (CRC)*				
Urine from CRC patients and healthy controls	HPLC-MS/MS method based on D-BPCl probe	D-2-AABA, D-Ala, D-Asn, D-Gln, D-Ile, D-Leu, D-Met, D-Phe, D-Ser, D-Thr, D-Trp, D-Tyr, D-Val, D-Cit	A diagnostic biomarker model using D-AABA, D-Ala, D-Gln, and D-Ser achieved high accuracy (98.3% sensitivity and 96.8% specificity) D-Met decreased in CRC patients > 50 years old, while D/L-Gln was higher in stage IV CRC patients compared to stage I patients D-amino acids can represent potential biomarkers for early CRC detection	[20]
HCT116 colorectal cancer cells and NCM460 normal intestinal epithelial cells	HPLC-MS/MS method based on D-BPCl probe	D-Ala, D-Gln, D-Leu, D-Met, D-Phe, D-Ser, D-Trp, D-Tyr	The intra- and extracellular L/D-amino acid metabolism differs significantly between NCM460 and HCT116 cells Exogenous D-amino acid addition had no significant effect on normal cell proliferation, but inhibited cancer cell proliferation	[50]
*Glioblastoma (GBM)*				
Resected tumour and non-tumour tissues, blood, and urine from patients with primary gliomas and healthy controls	2D-HPLC-FLD	D-Ser, D-Ala, D-Asn, D-Pro	Urinary D-Asn levels were significantly lower in GBM patients and increased after tumour removal (the decrease was confirmed in a GBM mouse model) Urinary D-Asn testing could enable non-invasive, early-stage glioma detection in routine medical settings	[57]
*Breast cancer (BC)*				
MCF-7 breast cancer cells and MCF-10A non-tumorigenic breast cells	HPLC-MS/MS with chiral separation	D-Asp, D-Ser	D-Ser and D-Asp levels were significantly elevated in breast cancer cells compared to non-tumorigenic cells D-amino acids may act as exchange currency, allowing for the uptake of essential and low-abundance nonessential amino acids A proposed malignancy indicator based on specific L- and D-amino acid relative levels could enable fast and non-invasive BC detection	[90]

Abbreviations: 2D, two dimensional; D-BPCl, 1-benzoyl-pyrrolidine-2-carboxylic acid 5-chloro-2-formyl-phenyl ester; DAAO, D-amino acid oxidase; FLD, fluorescence detector; HPLC, high-performance liquid chromatography; MS, mass spectrometry; MS/MS, tandem mass spectrometry; RP, reverse phase; SERS, surface-enhanced Raman scattering.

## Data Availability

No new data were created or analysed in this study. Data sharing is not applicable to this article.
